# Supplementary material for: Southwestern national park service employee risk, knowledge, and concern for triatomine exposure: A qualitative analysis using a novel knowledge, attitudes, and practices survey
Source: PLoS Negl Trop Dis. 2022 Sep 1;16(9):e0010744. doi: 10.1371/journal.pntd.0010744 (PMC9473629; doi:10.1371/journal.pntd.0010744)
Supplement: S1 File — (DOCX) [file pntd.0010744.s001.docx]

**Supporting Information**

**S1 File. Knowledge, attitudes, and practices survey.**

**Consent Page**

We are asking you to participate in a research study titled “Assessing the Knowledge, Attitudes, and Practices and the Risk of Triatomine Exposure of National Park Service Employees in Park Housing.” This study is being led by Antonio Alvarado, from the Department of Entomology at Cornell University and is an initiative of the CDC-funded Northeast Regional Center for Excellence in Vector-Borne Diseases. The Faculty Advisor for this study is Dr. Laura Harrington, from the Department of Entomology at Cornell University.

**What the study is about**

The purpose of this research study is to identify the knowledge, attitudes, and practices regarding Chagas disease of National Park Service employees. You were invited to participate in our study because you work in a southwestern national park of interest.

**What we will ask you to do**

We will ask you to complete this survey questionnaire truthfully and to the best of your ability. While taking the survey, please do not use any aids to answer the questions. The survey will take about 10 minutes of your time. In order for the survey results to be useful to our study team, we need you to answer all questions in the survey.

**Risks and discomforts**

We do not anticipate any risks from participating in this research.

**Benefits**

While you will not receive any direct benefits from participating in this research study, information from this study will help the National Park Service address the risk of Chagas disease in park housing.

**Compensation for participation**

There is no compensation for participation in this research.

**Privacy/Confidentiality/Data Security**

Your responses will be anonymous and the researcher will not collect any identifying personal information about you. Your survey answers will be stored in a password protected electronic format. We anticipate that your participation in this survey presents no greater risk than everyday use of the Internet.

**Sharing De-identified Data Collected in this Research**

De-identified data from this study may be shared with the research community at large to advance science and health. We will remove or code any personal information that could identify you before files are shared with other researchers to ensure that, by current scientific standards and known methods, no one will be able to identify you from the information we share. Despite these measures, we cannot guarantee anonymity of your personal data.

**Taking part is voluntary**

Your participation in this survey is voluntary. You may refuse to participate before the study begins or discontinue at any time. However, in order to participate, you must answer the required questions in this survey. The results of the survey will not be useful to us if you do not answer the required questions and will be less useful if you do not answer all of the questions. If you do not want to answer all of the questions, you may decline to participate now.

**If you have questions**

The main researcher conducting this study is Antonio Alvarado, a graduate student at Cornell University. Please ask any questions you have now at aa2757@cornell.edu. If you have questions later, you may contact Antonio via that email address or by telephone at 610-657-2043.  If you have any questions or concerns regarding your rights as a subject in this study, you may contact the Cornell University Institutional Review Board (IRB) for Human Participants at 607-255-5138 or access their website at http://www.irb.cornell.edu. You may also report your concerns or complaints anonymously through Ethicspoint online at www.hotline.cornell.edu or by calling toll free at 1-866-293-3077. Ethicspoint is an independent organization that serves as a liaison between the University and the person bringing the complaint so that anonymity can be ensured.

**Clicking on the "Agree" button indicates that:  You have read the above information, you voluntarily agree to participate, and you are 18 years of age or older.**

- Agree
- Disagree

**Demographics Part 1**

1. Which National Park unit do you work in?

- Big Bend National Park
- Chiricahua National Monument
- Coronado National Memorial
- Fort Bowie National Historic Site
- Organ Pipe Cactus National Monument
- Saguaro National Park
- Other
- Prefer not to answer

1. Do you live in NPS housing?

- Yes
- No
- Prefer not to answer

1. What best describes your length of employment with the National Park Service in years?

- < 3
- 3-10
- > 10
- Prefer not to answer

1. Which best describes the average amount of hours you work outdoors each week?

- None
- < 20
- 20-40
- > 40
- Prefer not to answer

**Knowledge Section**

The next few questions will ask you about your knowledge of Chagas disease. Please do not look up answers.

1. How familiar are you with Chagas disease signs and symptoms?

- None
- < 20
- 20-40
- > 40
- Prefer not to answer

1. Chagas disease is caused by a virus.

- Yes
- No
- Not sure

1. What are the potential symptoms of early infection with Chagas disease?

- Flu-like symptoms (fever, body aches, fatigue, and headache)
- Swelling around the eye
- Loss of appetite
- No symptoms
- All of the above
- None of the above
- Not sure

1. What are the potential long-term health effects of chronic infection with Chagas disease? (Select one option)

- Heart problems
- Digestive problems
- Both are potential long-term health effects
- None of the above
- Not sure

1. Dogs can get sick from Chagas disease

- Yes
- No
- Not sure

1. How is Chagas disease effectively treated? (Select one option)

- Anti-parasitic medication
- Anti-parasitic medication, but it is not approved in the United States
- Natural therapy (acupuncture, herbal remedies)
- Chagas disease cannot be treated
- None of the above

1. How can you get Chagas disease? (Select all that apply)

- Insect bite
- Mother-to-baby (congenital)
- Contaminated food or drink (oral)
- Blood transfusions
- Organ transplants
- None of the above
- Not sure

1. Which of the choices below show a triatomine bug (also known as a kissing bug)? (Select one option)

 
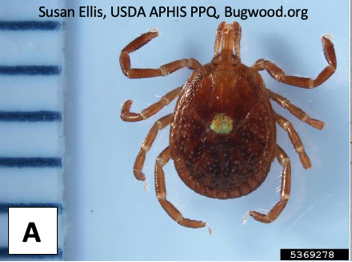

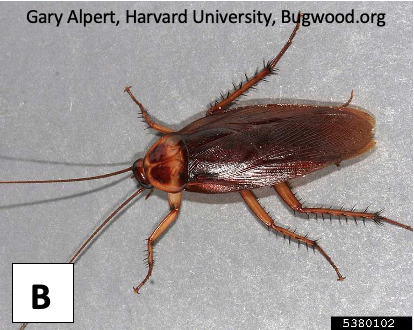


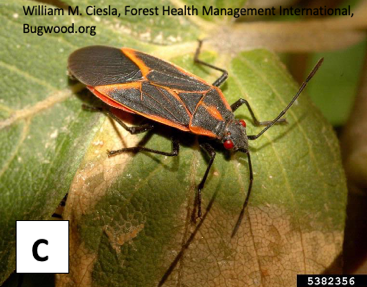

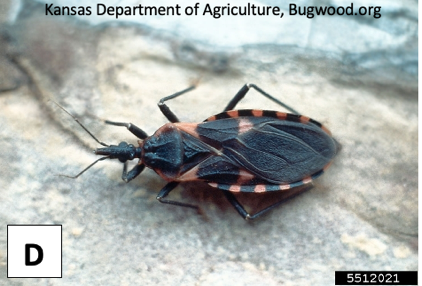

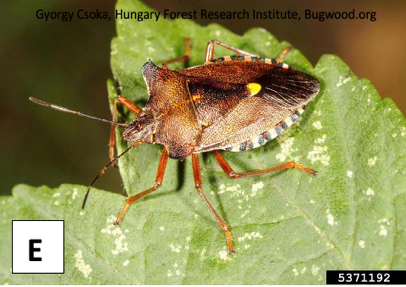


- A
- B
- C
- D
- E
- None of the above
- Not sure

1. Triatomine bugs transmit the disease agent that causes Chagas disease through their saliva.

- Yes
- No
- Not sure

1. What time of day are humans most at risk for being bitten by a triatomine bug? (select one option)

- Morning
- Afternoon
- Night
- Not sure

1. The presence of animals like pack rats, raccoons, and skunks are important factors for human risk of Chagas disease in the United States.

- Yes
- No
- Not sure

1. All triatomine bugs are infected with the parasite that causes Chagas disease.

- Yes
- No
- Not sure

This concludes the knowledge section. Please do not look at the following pages until you answer the previous questions and please do not change any answers.

**Attitudes Section**

Chagas disease is caused by the parasite *Trypanosoma cruzi*. The parasite is spread through the feces of infected triatomine bugs (also known as kissing bugs), which bite humans at night. Several animals maintain the parasite in their blood such as pack rats, raccoons, skunks, and coyotes, which can infect bugs. It can also be spread through food or drink contaminated with triatomine feces, blood transfusions, organ transplants, and mother-to-baby. While rare, there are cases of locally-acquired Chagas diseases in humans and dogs in the United States. 

Please let us know your attitudes regarding the following sentences about Chagas disease and triatomine bugs.

1. Chagas disease has negatively changed the way I feel about working with the National Park Service.

- Strongly agree
- Somewhat agree
- Neither agree nor disagree
- Somewhat disagree
- Strongly disagree

1. Chagas disease is a serious illness in the surrounding area.

- Strongly agree
- Somewhat agree
- Neither agree nor disagree
- Somewhat disagree
- Strongly disagree

1. I am at risk for getting Chagas disease while working with the National Park Service.

- Strongly agree
- Somewhat agree
- Neither agree nor disagree
- Somewhat disagree
- Strongly disagree

1. I am confident that I can correctly identify a triatomine bug.

- Strongly agree
- Somewhat agree
- Neither agree nor disagree
- Somewhat disagree
- Strongly disagree

1. If I am bitten by a triatomine bug, I will seek medical advice.

- Strongly agree
- Somewhat agree
- Neither agree nor disagree
- Somewhat disagree
- Strongly disagree

1. Triatomine bug control is important to me.

- Strongly agree
- Somewhat agree
- Neither agree nor disagree
- Somewhat disagree
- Strongly disagree

**Practices Section**

Thank you for answering the previous questions! The next questions ask about your personal practices and behaviors as they relate to potential triatomine bug exposure and Chagas disease.

1. How often do you take the following actions to reduce the chance of being bitten by a triatomine bug?
   1. I turn off inside and outside lights at night.
      - 1. Never
        2. Rarely
        3. Sometimes
        4. Often
        5. Always
   2. I have cracks or crevices in my house filled.
      - 1. Never
        2. Rarely
        3. Sometimes
        4. Often
        5. Always
   3. I make sure that my window screens have no rips or openings.
      - 1. Never
        2. Rarely
        3. Sometimes
        4. Often
        5. Always
   4. I close windows and doors at night.
      - 1. Never
        2. Rarely
        3. Sometimes
        4. Often
        5. Always
   5. I use air-conditioning.
      - 1. Never
        2. Rarely
        3. Sometimes
        4. Often
        5. Always
   6. I keep wood piles at least 10 feet away from my house
      - 1. Never
        2. Rarely
        3. Sometimes
        4. Often
        5. Always
   7. I remove debris from my yard (e.g. sticks, leaves)
      - 1. Never
        2. Rarely
        3. Sometimes
        4. Often
        5. Always
   8. I remove or cover food/trash sources (e.g. trash cans, pet food, compost piles)
      - 1. Never
        2. Rarely
        3. Sometimes
        4. Often
        5. Always
   9. I do not place storage containers along the sides of my house
      - 1. Never
        2. Rarely
        3. Sometimes
        4. Often
        5. Always
2. I get my information on triatomine bugs from: (Select all that apply)

- Centers for Disease Control and Prevention (CDC)
- State health department
- Academic institution (e.g. Texas A&M, University of Arizona, etc.)
- Park safety officer/NPS biologist
- Word of mouth
- Social media (e.g. Facebook, Twitter, Instagram, etc.)
- Other
- I do not look up information on triatomine bugs

1. If I am bitten by a triatomine bug, I will: (Select all that apply):

- Visit my primary care provider
- Visit an infectious disease specialist
- Visit the emergency room
- Contact park safety officer/NPS office
- Submit the bug for testing
- Stay at home
- Only seek medical care if I am sick
- Other

1. What control measures do you currently use to reduce the number of triatomine bugs in your house: (Select all that apply)

- Chemical pesticide
- Natural pesticide
- Sticky traps
- Bed nets
- Reduce outdoor lighting
- Create vegetation/debris free zone around the foundation of my home
- Other
- I do not take any actions to reduce triatomine bugs in my home

1. What control measures would you consider using to reduce the number of triatomine bugs in your house: (Select all that apply)

- Chemical pesticide
- Natural pesticide
- Sticky traps
- Bed nets
- Reduce outdoor lighting
- Create vegetation/debris free zone around the foundation of my home
- Other
- I would not consider taking any actions to reduce triatomine bugs in my home

**Triatomine and Small Mammal Exposure**

Thank you for answering the previous questions! The next questions ask about your personal experience with triatomine bugs, small mammals, and housing.

1.
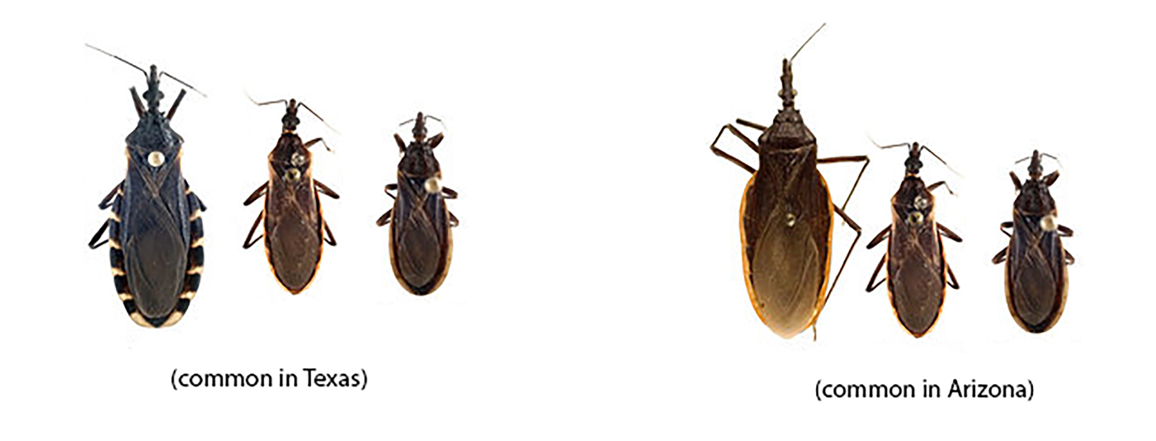
Have you seen any of these bugs during your employment with the National Park Service?

- Yes
- No
- Not sure

1. Have you found any of these bugs within your home during your employment with the National Park Service?

- Yes
- No
- Not sure

1. I have had unexplained insect bites while sleeping.

- Yes
- No
- Not sure

1. Where have you been bitten before on your body? (Select all that apply)

- Face
- Arm
- Hand
- Leg
- Foot
- Neck
- Other

1. How many times a year do you have unexplained insect bites?

- 0
- 1-5
- 6-10
- > 10

1. I have a pet.

- Yes
- No

1. What type of pet do you have?

- Dog
- Cat
- Other

1. Have you ever found triatomine bugs on your pet, in your pet's bedding, or in places where pet spends time?

- Yes
- No
- Not sure

1. I have seen small animals or rodents in or very near my home.

- Yes
- No
- Not sure

1. Where have you seen small animals or rodents? (Select all that apply)

- Crawl space
- Garage/Carport
- Attic
- Kitchen
- Sleeping quarters
- Living quarters
- Yard
- Other

1. On a scale of 1 to 10, how are you satisfied with the standard of insect control in your housing? (1 is completely dissatisfied, 5 is neither satisfied nor dissatisfied, and 10 is completely satisfied) (Select one option)

- 1 (completely dissatisfied)
- 2
- 3
- 4
- 5 (neither satisfied nor dissatisfied)
- 6
- 7
- 8
- 9
- 10 (completely satisfied)

**Demographics Part 2**

1. What is your age range in years?

- 18-30
- 31-50
- > 50
- Prefer not to answer

1. What is your gender?

- Male
- Female
- Non-binary/Other
- Prefer not to answer

1. What is your race/ethnicity?

- Black or African American
- Hispanic/Latinx
- American Indian or Alaska Native
- Asian
- Native Hawaiian or Pacific Islander
- White
- Multi-ethnic/Other Race/Ethnicity
- Prefer not to answer

1. What is the highest level of education you have attained?

- High School or Less
- College Student
- College Graduate
- Graduate/Higher
- Prefer not to answer

We thank you for your time spent taking this survey! For more information on Chagas disease and triatomine bugs please visit the CDC's website: https://www.cdc.gov/parasites/chagas/gen_info/detailed.html#intro
