# Supplementary material for: Southwestern national park service employee risk, knowledge, and concern for triatomine exposure: A qualitative analysis using a novel knowledge, attitudes, and practices survey
Source: PLoS Negl Trop Dis. 2022 Sep 1;16(9):e0010744. doi: 10.1371/journal.pntd.0010744 (PMC9473629; doi:10.1371/journal.pntd.0010744)
Supplement: S1 Table — (DOCX) [file pntd.0010744.s002.docx]

**Supporting Information**

**S1 Table. KAP survey responses.**

| **Demographics and General Information** | | | |
| --- | --- | --- | --- |
|  | | Number of responses (%) | |
| National park | Big Bend National Park | 29 (32.6) | |
|  | Southeast Arizona Group | 17 (19.1) | |
|  | Organ Pipe National Monument | 21 (23.6) | |
|  | Saguaro National Park | 22 (24.7) | |
| Age | 18-30 | 10 (11.2) | |
|  | 31-50 | 23 (25.8) | |
|  | > 50 | 25 (28.1) | |
|  | Prefer not to answer | 2 (2.2) | |
|  | NA | 29 (32.6) | |
| Gender | Male | 36 (40.4) | |
|  | Female | 20 (22.5) | |
|  | Non-binary/other | 1 (1.1) | |
|  | Prefer not to answer | 3 (3.4) | |
|  | NA | 29 (32.6) | |
| Race/ethnicity | Hispanic/Latinx | 8 (9.1) | |
|  | Native Hawaiian or Pacific Islander | 1 (1.1) | |
|  | White | 44 (49.4) | |
|  | Multi-ethnic/Other Race/ethnicity | 2 (2.2) | |
|  | Prefer not to answer | 5 (5.6) | |
|  | NA | 29 (32.6) | |
| Education level | High school or less | 4 (4.5) | |
|  | College student | 6 (6.7) | |
|  | College graduate | 28 (31.5) | |
|  | Graduate/higher | 20 (22.5) | |
|  | Prefer not to answer | 2 (2.2) | |
|  | NA | 29 (32.6) | |
| Lives in NPS housing | Yes | 49 (55.1) | |
|  | No | 40 (44.9) | |
| Length of employment with NPS in years | < 3 | 15 (16.8) | |
|  | 3-10 | 17 (19.1) | |
|  | > 10 | 28 (31.5) | |
|  | NA | 29 (32.6) | |
| Average amount of hours worked outdoors each week | None | 9 (10.1) | |
|  | < 20 | 39 (43.8) | |
|  | 3-10 | 31 (34.8) | |
|  | > 10 | 9 (10.1) | |
|  | Prefer not to answer | 1 (1.1) | |
| General satisfaction with standard of housing conditions on a 1-10 scale | 1 | 3 (3.4) | |
|  | 2 | 1 (1.1) | |
|  | 5 | 14 (15.7) | |
|  | 6 | 6 (6.7) | |
|  | 7 | 4 (4.5) | |
|  | 8 | 13 (14.6) | |
|  | 9 | 10 (11.2) | |
|  | 10 | 9 (10.1) | |
|  | NA | 29 (32.6) | |
| **Triatomine and Small Mammal Exposure** | | | |
| Have you seen any triatomine bugs during your NPS employment? | Yes | 60 (67.4) | |
| Have you found any triatomine bugs within your home during your NPS employment? | Yes | 29 (48.3) | |
| Have you had unexplained insect bites while sleeping? | Yes | 32 (36.0) | |
| How many times a year do you have unexplained insect bites? | 1-5 | 23 (71.9) | |
|  | 6-10 | 7 (21.9) | |
|  | > 10 | 2 (6.2) | |
| Where have you been bitten before on your body? | Face | 7 (21.9) | |
|  | Arm | 21 (65.6) | |
|  | Hand | 5 (15.6) | |
|  | Leg | 27 (84.4) | |
|  | Foot | 11 (34.4) | |
|  | Neck | 5 (15.6) | |
|  | Other | 8 (25.0) | |
| Do you have a pet? | Yes | 49 (55.1) | |
| What type of pet do you have? | Dog | 31 (63.3) | |
|  | Cat | 18 (36.7) | |
| Have you ever found triatomine bugs on your pet, in your pet's bedding, or in places where your pet spends time? | Yes | 1 (2.0) | |
| Have you seen small animals or rodents in or very near your home? | Yes | 67 (75.3) | |
| Where have you seen small animals or rodents? | Crawl space | 13 (19.4) | |
|  | Garage/carport | 26 (38.8) | |
|  | Attic | 9 (13.4) | |
|  | Kitchen | 19 (28.3) | |
|  | Sleeping quarters | 5 (7.5) | |
|  | Living quarters | 13 (19.4) | |
|  | Yard | 62 (92.5) | |
|  | Other | 3 (4.5) | |
| **Knowledge** | | | |
| How familiar are you with Chagas disease signs and symptoms? | Very familiar | 5 (5.6) | |
|  | Familiar | 12 (13.5) | |
|  | Somewhat familiar | 42 (47.2) | |
|  | Not familiar at all | 29 (32.6) | |
|  | NA | 1 (1.1) | |
| Chagas disease is caused by a virus. | Yes | 11 (12.4) | |
|  | No | 40 (44.9) | |
|  | Not sure | 37 (41.6) | |
|  | NA | 1 (1.1) | |
| What are the potential symptoms of early infection with Chagas disease? | Flu-like symptoms | 20 (22.5) | |
|  | Swelling around the eye | 5 (5.6) | |
|  | No symptoms | 3 (3.4) | |
|  | All of the above | 24 (27.0) | |
|  | Not sure | 36 (40.4) | |
|  | NA | 1 (1.1) | |
| What are the potential long-term health effects of chronic infection with Chagas disease? | Heart problems | 19 (21.3) | |
|  | Both are potential long-term health effects | 30 (33.7) | |
|  | NA | 40 (44.9) | |
| Dogs can get sick from Chagas disease. | Yes | 38 (42.7) | |
|  | Not sure | 50 (56.2) | |
|  | NA | 1 (1.1) | |
| How is Chagas disease effectively treated? | Anti-parasitic medication | 26 (29.2) | |
|  | Anti-parasitic medication, but it is not approved in the United States | 1 (1.1) | |
|  | Not sure | 56 (62.9) | |
|  | NA | 6 (6.7) | |
| How can you get Chagas disease? (select all that apply) | Insect bite | 76 (85.4) | |
|  | Mother-to-baby | 10 (11.2) | |
|  | Contaminated food or drink | 7 (7.9) | |
|  | Blood transfusions | 12 (13.5) | |
|  | Organ transplants | 8 (9.1) | |
|  | NA | 12 (13.5) | |
| Triatomine identification: Which of the choices show a triatomine bug? | Lone star tick | 1 (1.) | |
|  | Boxelder bug | 3 (3.4) | |
|  | Stink bug | 1 (1.1) | |
|  | Triatomine bug | 76 (85.4) | |
|  | None of the above | 1 (1.1) | |
|  | Not sure | 6 (6.7) | |
|  | NA | 1 (1.1) | |
| Triatomine bugs transmit the disease agent that causes Chagas disease through their saliva. | Yes | 48 (53.9) | |
|  | No | 25 (28.1) | |
|  | Not sure | 15 (16.8) | |
|  | NA | 1 (1.1) | |
| What time of day are humans most at risk for being bitten by a triatomine bug? | Night | 74 (83.2) | |
|  | Not sure | 14 (15.7) | |
|  | NA | 1 (1.1) | |
| The presence of animals like pack rats, raccoons, and skunks are important factors for human risk of Chagas disease in the United States. | Yes | 44 (49.4) | |
|  | No | 5 (5.6) | |
|  | Not sure | 39 (43.8) | |
|  | NA | 1 (1.1) | |
| All triatomine bugs are infected with the parasite that causes Chagas disease. | Yes | 7 (7.9) | |
|  | No | 59 (66.3) | |
|  | Not sure | 22 (24.7) | |
|  | NA | 1 (1.1) | |
| **Attitudes** | | | |
| Chagas disease has negatively changed the way I feel about working with the National Park Service. | Strongly agree | 5 (5.6) | |
|  | Somewhat agree | 8 (9.0) | |
|  | Neither agree nor disagree | 18 (20.2) | |
|  | Somewhat disagree | 8 (9.0) | |
|  | Strongly disagree | 50 (56.2) | |
| Chagas disease is a serious illness in the surrounding area. | Strongly agree | 11 (12.3) | |
|  | Somewhat agree | 21 (23.6) | |
|  | Neither agree nor disagree | 36 (40.4) | |
|  | Somewhat disagree | 13 (14.6) | |
|  | Strongly disagree | 8 (9.1) | |
| I am at risk for getting Chagas disease while working with the National Park Service. | Strongly agree | 13 (14.6) | |
|  | Somewhat agree | 39 (43.8) | |
|  | Neither agree nor disagree | 19 (21.3) | |
|  | Somewhat disagree | 12 (13.5) | |
|  | Strongly disagree | 6 (6.7) | |
| I am confident that I can correctly identify a triatomine bug. | Strongly agree | 30 (33.7) | |
|  | Somewhat agree | 34 (38.2) | |
|  | Neither agree nor disagree | 13 (14.6) | |
|  | Somewhat disagree | 7 (7.9) | |
|  | Strongly disagree | 5 (5.6) | |
| If I am bitten by a triatomine bug, I will seek medical advice. | Strongly agree | 42 (47.2) | |
|  | Somewhat agree | 17 (19.1) | |
|  | Neither agree nor disagree | 15 (16.8) | |
|  | Somewhat disagree | 13 (14.6) | |
|  | Strongly disagree | 2 (2.2) | |
| Triatomine bug control is important to me. | Strongly agree | 53 (59.5) | |
|  | Somewhat agree | 21 (23.6) | |
|  | Neither agree nor disagree | 10 (11.2) | |
|  | Somewhat disagree | 4 (4.5) | |
|  | Strongly disagree | 1 (1.1) | |
| I approve of using chemical pesticides on or near my home to control triatomine bugs. | Strongly agree | 21 (23.6) | |
|  | Somewhat agree | 12 (13.5) | |
|  | Neither agree nor disagree | 13 (14.6) | |
|  | Somewhat disagree | 9 (10.1) | |
|  | Strongly disagree | 5 (5.6) | |
|  | NA | 29 (32.6) | |
| The National Park Service is doing their best to prevent Chagas disease. | Strongly agree | 1 (1.1) | |
|  | Somewhat agree | 10 (11.2) | |
|  | Neither agree nor disagree | 34 (38.2) | |
|  | Somewhat disagree | 9 (10.1) | |
|  | Strongly disagree | 6 (6.7) | |
|  | NA | 29 (32.6) | |
| **Practices** | | | |
| How often do you take the following actions to reduce the chance of being bitten by a triatomine bug? | Turn off inside and outside lights at night. | Never | 4 (4.5) |
|  |  | Rarely | 1 (1.1) |
|  |  | Sometimes | 11 (12.3) |
|  |  | Often | 28 (31.5) |
|  |  | Always | 45 (50.6) |
|  | Have cracks or crevices filled. | Never | 14 (15.7) |
|  |  | Rarely | 11 (12.3) |
|  |  | Sometimes | 21 (23.6) |
|  |  | Often | 30 (33.7) |
|  |  | Always | 13 (14.6) |
|  | Make sure window screens have no rips or openings. | Never | 8 (9.1) |
|  |  | Rarely | 12 (13.5) |
|  |  | Sometimes | 20 (22.5) |
|  |  | Often | 28 (31.5) |
|  |  | Always | 21 (23.6) |
|  | Close windows and doors at night. | Never | 3 (3.4) |
|  |  | Rarely | 7 (7.9) |
|  |  | Sometimes | 19 (21.3) |
|  |  | Often | 15 (16.8) |
|  |  | Always | 45 (50.6) |
|  | Use air-conditioning. | Never | 7 (7.9) |
|  |  | Rarely | 6 (6.7) |
|  |  | Sometimes | 22 (24.7) |
|  |  | Often | 28 (31.5) |
|  |  | Always | 26 (29.2) |
|  | Keep wood piles at least 10 feet away from my house. | Never | 2 (2.2) |
|  |  | Rarely | 1 (1.1) |
|  |  | Sometimes | 10 (11.2) |
|  |  | Often | 21 (23.6) |
|  |  | Always | 53 (59.5) |
|  |  | NA | 2 (2.2) |
|  | Remove debris from my yard. | Never | 10 (11.2) |
|  |  | Rarely | 9 (10.1) |
|  |  | Sometimes | 29 (32.6) |
|  |  | Often | 19 (21.3) |
|  |  | Always | 21 (23.6) |
|  |  | NA | 1 (1.1) |
|  | Remove or cover food/trash sources. | Never | 1 (1.1) |
|  |  | Rarely | 5 (5.6) |
|  |  | Sometimes | 12 (13.5) |
|  |  | Often | 24 (27.0) |
|  |  | Always | 47 (52.8) |
|  | Do not place storage boxes or containers along the sides of my house. | Never | 7 (7.9) |
|  |  | Rarely | 5 (5.6) |
|  |  | Sometimes | 16 (18.0) |
|  |  | Often | 20 (22.5) |
|  |  | Always | 39 (43.8) |
|  |  | NA | 2 (2.2) |
| Where do you get your information on triatomine bugs from? (select all that apply) | Centers for Disease Control and Prevention | 32 (35.9) | |
|  | State health department | 11 (12.3) | |
|  | Academic institution | 22 (24.7) | |
|  | Park safety officer/NPS biologist | 51 (57.3) | |
|  | Word of mouth | 28 (31.5) | |
|  | Social media | 5 (5.6) | |
|  | Other | 11 (12.3) | |
|  | I do not look up information on triatomine bugs | 16 (18.0) | |
| If you are bitten by a triatomine bug, what would you do? (select all that apply) | Visit my primary care provider | 43 (48.3) | |
|  | Visit an infectious disease specialist | 6 (6.7) | |
|  | Visit the emergency room | 3 (3.4) | |
|  | Contact park safety officer/NPS office | 40 (44.9) | |
|  | Submit the bug for testing | 32 (35.9) | |
|  | Stay at home | 2 (2.2) | |
|  | Only seek medical care if I am sick | 34 (38.2) | |
|  | Other | 4 (4.5) | |
| What control measures do you currently use to reduce the number of triatomine bugs in your house? (select all that apply) | Chemical pesticide | 11 (12.3) | |
|  | Natural pesticide | 4 (4.5) | |
|  | Sticky traps | 13 (14.6) | |
|  | Bed nets | 1 (1.1) | |
|  | Reduce outdoor lighting | 54 (60.7) | |
|  | Create a vegetation/debris free zone around the foundation of my home | 29 (32.6) | |
|  | Other | 7 (7.9) | |
|  | None | 27 (30.3) | |
| What control measures would you consider using to reduce the number of triatomine bugs in your house? (select all that apply) | Chemical pesticide | 39 (43.8) | |
|  | Natural pesticide | 65 (73.0) | |
|  | Sticky traps | 49 (55.0) | |
|  | Bed nets | 21 (23.6) | |
|  | Reduce outdoor lighting | 55 (61.8) | |
|  | Create a vegetation/debris free zone around the foundation of my home | 62 (69.7) | |
|  | Other | 12 (13.5) | |
|  | None | 5 (5.6) | |
