# Supplementary material for: Southwestern national park service employee risk, knowledge, and concern for triatomine exposure: A qualitative analysis using a novel knowledge, attitudes, and practices survey
Source: PLoS Negl Trop Dis. 2022 Sep 1;16(9):e0010744. doi: 10.1371/journal.pntd.0010744 (PMC9473629; doi:10.1371/journal.pntd.0010744)
Supplement: S2 Table — (DOCX) [file pntd.0010744.s003.docx]

**Supporting Information**

**S2 Table. Principle component analysis for attitudes items.**

| Items | Factor Loadings for Perceived Anxiety Regarding CD | Factor Loadings for Personal Agency to Address CD Risk |
| --- | --- | --- |
| CD negatively affects my feelings of working for the NPS | 0.72 |  |
| CD is a serious illness in the local area | 0.73 |  |
| I am at risk for getting CD while working for the NPS | 0.84 |  |
| I will seek medical advice following a triatomine bug bite. |  | 0.87 |
| Triatomine bug control is important to me. |  | 0.75 |
| Sum of Square Loadings | 1.77 | 1.36 |
| Proportion Variance | 0.35 | 0.27 |
| RMSR | 0.16 | |
| Empirical Chi-Square | 42.86 (p<0.0001) | |
