# Supplementary material for: Southwestern national park service employee risk, knowledge, and concern for triatomine exposure: A qualitative analysis using a novel knowledge, attitudes, and practices survey
Source: PLoS Negl Trop Dis. 2022 Sep 1;16(9):e0010744. doi: 10.1371/journal.pntd.0010744 (PMC9473629; doi:10.1371/journal.pntd.0010744)
Supplement: Attachment — Submitted filename: Reviewers_response.docx [file pntd.0010744.s004.docx]

Dear Editor,

we have responded to reviewer 2’s comments below.

Dear Mr. Alvarado,

Thank you very much for submitting your manuscript "Southwestern National Park Service Employee Risk, Knowledge, and Concern for Triatomine Exposure: A Qualitative Analysis Using a Novel Knowledge, Attitudes, and Practices Survey" for consideration at PLOS Neglected Tropical Diseases. As with all papers reviewed by the journal, your manuscript was reviewed by members of the editorial board and by several independent reviewers. In light of the vastly differing reviews (below this email), we would like to invite the resubmission of a significantly-revised version that takes into account the reviewers' comments.

We cannot make any decision about publication until we have seen the revised manuscript and your response to the reviewers' comments. Your revised manuscript is also likely to be sent to reviewers for further evaluation.

When you are ready to resubmit, please upload the following:

[1] A letter containing a detailed list of your responses to the review comments and a description of the changes you have made in the manuscript. Please note while forming your response, if your article is accepted, you may have the opportunity to make the peer review history publicly available. The record will include editor decision letters (with reviews) and your responses to reviewer comments. If eligible, we will contact you to opt in or out.

[2] Two versions of the revised manuscript: one with either highlights or tracked changes denoting where the text has been changed; the other a clean version (uploaded as the manuscript file).

Important additional instructions are given below your reviewer comments.

Please prepare and submit your revised manuscript within 60 days. If you anticipate any delay, please let us know the expected resubmission date by replying to this email. Please note that revised manuscripts received after the 60-day due date may require evaluation and peer review similar to newly submitted manuscripts.

Thank you again for your submission. We hope that our editorial process has been constructive so far, and we welcome your feedback at any time. Please don't hesitate to contact us if you have any questions or comments.

Sincerely,

Rhoel Ramos Dinglasan

Associate Editor

PLOS Neglected Tropical Diseases

Walderez Dutra

Deputy Editor

PLOS Neglected Tropical Diseases

***********************

Reviewer's Responses to Questions

<b>Key Review Criteria Required for Acceptance?</b></br></br> As you describe the new analyses required for acceptance, please consider the following:</br></br>

<b>Methods</b></br></br>

-Are the objectives of the study clearly articulated with a clear testable hypothesis stated?</br> -Is the study design appropriate to address the stated objectives?</br> -Is the population clearly described and appropriate for the hypothesis being tested?</br> -Is the sample size sufficient to ensure adequate power to address the hypothesis being tested?</br> -Were correct statistical analysis used to support conclusions?</br> -Are there concerns about ethical or regulatory requirements being met?</br></br>

Reviewer #1: Yes

Reviewer #2: Are the objectives of the study clearly articulated with a clear testable hypothesis stated?

No, the methodology used has many deficiencies and there was no control of biases derived from the methodology.

RESPONSE: Without out details on the perceived deficiencies, we cannot respond.

-Is the study design appropriate to address the stated objectives the study design only gives it a descriptive scope, the lack of randomization and a possible imposition on the participants to increase the number of participants in the study, can invalidate the results. Additionally, the methodology is not adequate to compare between groups.

RESPONSE: Our research goals and methodology are clearly stated and are not simply “descriptive”. This type of survey research does not call for randomization, as our goal was to obtain a representative sample of a small, targeted population of NPS employees. If anything, since we have a high response rate from this group, our results are more robust than a random selection. Our methodology is adequate to compare groups.

Rev 2 implied in several places that our approach was biased and “imposed” or forced participants to contribute responses. We don’t understand how they reached this conclusion. We had an IRB reviewed study, and participation was voluntary and anonymous, so there was no way people were coerced to participate. The reviewer also does not state the specific biases we failed to address in our methodology, which is needed in order for us to respond to this critique.

-Is the sample size sufficient to ensure adequate power to address the hypothesis being tested?

the number of participants is adequate, however, it is not clear whether participation is entirely voluntary -Were correct statistical analysis used to support conclusions?

RESPONSE: We used an IRB reviewed study and participation was both voluntary and anonymous, so there was no way people were coerced to participate.

The authors perform routine statistical analyzes and those that are done in similar studies, however, it is better to adopt other methodologies such as semi-structured interviews, which allow qualitative analysis. Additionally, not using triatomine samplers does not allow the participant to differentiate between reduced hematophagous, predatory and phytophagous... This possible memory bias is key not controlled, it is key to have wrong results -Are there concerns about ethical or regulatory requirements being met?

there is no such concern,is ok

RESPONSE: We chose not to follow a qualitative methodology for this study, as our goal was to generalize our survey findings to the population of NPS employees at the targeted parks. This is not achievable through qualitative methods, which by their nature cannot provide generalizable data. We cannot understand the remainder of this comment.

<b>Results</b></br></br>

-Does the analysis presented match the analysis plan?</br> -Are the results clearly and completely presented?</br> -Are the figures (Tables, Images) of sufficient quality for clarity?</br></br>

Reviewer #1: Yes, the results were appropriate and rigourously collected.

Reviewer #2: -Does the analysis presented match the analysis plan?

Yes, they represent the analysis plan

-Are the results clearly and completely presented?

Yes, they are clearly present

-Are the figures (Tables, Images) of sufficient quality for clarity?

Table 1 it is innecesary

RESPONSE: We disagree, this information is important for readers to have.

At line 272, the authors said " NPS employees had low knowledge of CD and triatomine vectors". However, in the table 2 we found a differnt datas.. Correctly identified triatomine: 96,55%,82.35%, 76, 19%...

RESPONSE: The criterion Rev2 is referring to was just 1 of 10 used to evaluate knowledge regarding both triatomine vectors and Chagas disease. We describe in our methods how we developed a knowledge scale from several question items.

The question "The presence of animal reservoirs, such as pack rats, are important factors for human risk of CD". The authors have no evidence if this species plays a role as reservoirs in this ecosystem

RESPONSE: Pack rats are one of the most important species for kissing bugs in these settings, as we stated on lines 111-112) (eg <https://wwwnc.cdc.gov/eid/article/9/1/02-0217_article> )

--------------------

<b>Conclusions</b></br></br>

-Are the conclusions supported by the data presented?</br> -Are the limitations of analysis clearly described?</br> -Do the authors discuss how these data can be helpful to advance our understanding of the topic under study?</br> -Is public health relevance addressed?</br></br>

Reviewer #1: Yes, conclusions were appropriate based on the findings.

Reviewer #2: -Are the conclusions supported by the data presented?

no, the authors cannot be clear whether the outcome they evaluated (recognition of the vector) was not influenced by a reporting or recall bias. It is clear that the people evaluated live in areas with potential transmission, but there are other methodologies that allow to see if there is contact between people and triatomines and potential reservoirs in a particular environment.

-Are the limitations of analysis clearly described?

biases and controls were not reported

RESPONSE: What biases and controls? What methodologies? It was not our intent to directly measure contact between people and triatomines. Furthermore, we had several outcomes of importance generated through the survey questionnaire, only one of which was recognition of triatomine vectors as part of the knowledge section. Rather, we evaluated the knowledge of Chagas disease and kissing bugs, perceptions regarding Chagas disease risk, and behaviors regarding Chagas disease prevention in this KAP survey. Recall bias was unlikely to affect the recognition of a triatomine vector, as we showed respondents the image of several arthropods for real-time identification (our questionnaire is included in the supplemental files).

-Do the authors discuss how these data can be helpful to advance our understanding of the topic under study?

no, 203 / 5.000

RESPONSE: We cannot interpret this comment and therefore cannot respond.

Resultados de traducción

many of the conclusions of this study have been reported previously. currently, there are new methodologies and other types of approaches to know the risk that employees of NPS -Is public health relevance addressed?

RESPONSE: This is not correct, there are few studies in the US that address Chagas disease KAP and none that have been conducted among National Park Service employees. We’ve stated this in several places in the manuscript including lines 129-134, “address the gap in understanding CD knowledge and preventive practices among NPS employees by determining 1) the influence of NPS employee knowledge and attitudes on their triatomine prevention behaviors, 2) the triatomine prevention and control measures that NPS employees are currently using and willing to use, 3) self-reported human and triatomine exposure frequency, and (4) the risks to NPS employees.”

If the conclusions of this study have been reported elsewhere, perhaps the reviewer can share that information, because we can find no such published data.

Our conclusions are as follows (lines 58-63): “We detected high self-reported triatomine bug bite exposure in southwestern national parks. Additionally, NPS employees overall had low knowledge of CD, but greater knowledge of CD was not associated with better practices. Human emotions, behavior, and environmental factors are deeply rooted within the CD transmission cycle. This research adds to the growing body of literature on the CD knowledge, attitudes, and practices of a high-risk population in the US.”

Studies that visualize the potential risk of people living in areas with the presence of enzootic transmission in the United States are interesting, however, more robust approximations must be made. Unfortunately, the methodology used does not allow knowing this

RESPONSE: The review repeatedly states that we need to have more robust “approximations” and methods, but without a clear example of what Rev2 means, we cannot respond to the concern.

--------------------

<b>Editorial and Data Presentation Modifications?</b></br><br/> Use this section for editorial suggestions as well as relatively minor modifications of existing data that would enhance clarity. If the only modifications needed are minor and/or editorial, you may wish to recommend “Minor Revision” or “Accept”.

Reviewer #1: Accept

Reviewer #2: (No Response)

--------------------

<b>Summary and General Comments</b></br></br> Use this section to provide overall comments, discuss strengths/weaknesses of the study, novelty, significance, general execution and scholarship. You may also include additional comments for the author, including concerns about dual publication, research ethics, or publication ethics. If requesting major revision, please articulate the new experiments that are needed.

Reviewer #1: Well written article that contributes to the scientific literature.

Reviewer #2: These types of studies are important and necessary, however, the methodology adopted and the lack of bias control do not allow knowing the potential risk of infection. The results show that there is an apparent ignorance of the employees of the potential risk of vector and oral transmission. However, more appropriate outcomes (triatomine collection, photo taking, use of apps) are recommended. It is better to use semi-structured interviews with key informants and to carry out qualitative analyses.

RESPONSE: What bias are they referring to? What methodology was bad in their opinion? We used standard rigorous and reproducible approaches for KAP studies. Semi-structured interviews can provide rich qualitative data but only for a small number of individuals and cannot be generalized to the larger group of NPS employees at these parks.

--------------------

PLOS authors have the option to publish the peer review history of their article (<a href="<https://journals.plos.org/plosntds/s/editorial-and-peer-review-process#loc-peer-review-history>" target="_blank">what does this mean?</a>). If published, this will include your full peer review and any attached files.<br><br>

If you choose “no”, your identity will remain anonymous but your review may still be made public.<br><br>

<b>Do you want your identity to be public for this peer review?</b> For information about this choice, including consent withdrawal, please see our <a href="<https://www.plos.org/privacy-policy>" target="_blank">Privacy Policy</a>.

Reviewer #1: No

Reviewer #2: No

Figure Files:

While revising your submission, please upload your figure files to the Preflight Analysis and Conversion Engine (PACE) digital diagnostic tool, <https://pacev2.apexcovantage.com>. PACE helps ensure that figures meet PLOS requirements. To use PACE, you must first register as a user. Then, login and navigate to the UPLOAD tab, where you will find detailed instructions on how to use the tool. If you encounter any issues or have any questions when using PACE, please email us at [figures@plos.org](mailto:figures@plos.org).

Data Requirements:

Please note that, as a condition of publication, PLOS' data policy requires that you make available all data used to draw the conclusions outlined in your manuscript. Data must be deposited in an appropriate repository, included within the body of the manuscript, or uploaded as supporting information. This includes all numerical values that were used to generate graphs, histograms etc.. For an example see here: <http://www.plosbiology.org/article/info%3Adoi%2F10.1371%2Fjournal.pbio.1001908#s5>.

Reproducibility:

To enhance the reproducibility of your results, we recommend that you deposit your laboratory protocols in protocols.io, where a protocol can be assigned its own identifier (DOI) such that it can be cited independently in the future. Additionally, PLOS ONE offers an option to publish peer-reviewed clinical study protocols. Read more information on sharing protocols at <https://plos.org/protocols?utm_medium=editorial-email&utm_source=authorletters&utm_campaign=protocols>

__________________________________________________

In compliance with data protection regulations, you may request that we remove your personal registration details at any time. (Use the following URL: <https://www.editorialmanager.com/pntd/login.asp?a=r>). Please contact the publication office if you have any questions.
